# Are chiropractors in the uk primary healthcare or primary contact practitioners?: a mixed methods study

**DOI:** 10.1186/1746-1340-18-28

**Published:** 2010-10-27

**Authors:** Amanda R Jones-Harris

**Affiliations:** 1 Senior Lecturer Chiropractic Sciences, Department of Academic Affairs Anglo-European College of Chiropractic Bournemouth, Dorset, UK

## Abstract

**Background:**

One of the debates regarding the role of chiropractors is whether or not they should be considered as primary healthcare practitioners. Primary care is often used to describe chiropractic but without any definition of what is meant by the term. Primary healthcare itself has many definitions and this adds to the problem. Existing research literature, based mostly in the USA, suggests that the use of the title "primary healthcare professional" by chiropractors is central to the identity of the profession. It has also been suggested that the concept of primary care is misused by chiropractors because they have not examined the concept in detail and thus do not understand it. For the sake of quality of patient care and for the legitimacy of the profession, chiropractors in the UK need to agree on their healthcare role. This study aimed to examine the opinions of chiropractors towards the use of the term primary healthcare when applied to chiropractic practice within the UK.

**Methods:**

A sequential study of exploratory design was used; this model is characterised by an initial phase of qualitative data collection and analysis that precedes and informs the quantitative phase of data collection and analysis. In this study, interviews with members of chiropractic teaching faculty were used to inform the development of a questionnaire used to survey the opinions of chiropractors in the UK.

**Results:**

There was a general consensus of opinion that chiropractors are primary contact practitioners, who work in a primary healthcare setting and that to be able to fulfil this healthcare role, chiropractors must be able to diagnose patients and refer when required. Participants did not feel that chiropractors are able to treat all of the most common medical conditions that present in a primary healthcare setting.

**Conclusions:**

The findings of this study suggest that chiropractors in the UK view their role as one of a primary contact healthcare practitioner and that this view is held irrespective of the country in which they were educated or the length of time in practice.

Further research needs to be developed to evaluate the findings of the current study within a wider healthcare context. In particular the opinions of other healthcare professionals towards the role of chiropractors in healthcare, need to be examined in more detail.

## Background

Chiropractic is a healthcare profession concerned with the diagnosis, treatment and prevention of disorders of the musculoskeletal system, and the effects of these disorders on the nervous system and general health [1]. Chiropractic has evolved from a heterodox health system claiming to be a complete alternative to orthodox medicine to one of neuromusculoskeletal specialist complementary to general medical care [2].

Enactment of the Chiropractors Act in 2004 protects the title 'chiropractor' under British Law and legislation authorises primary contact practice, meaning that patients may consult a chiropractor directly without referral from another healthcare professional. Primary contact practice means that chiropractors have the right and a duty to perform a diagnosis [1].

One of the debates regarding the role of chiropractors is whether or not they should be considered as primary healthcare practitioners. Primary care is often used to describe chiropractic but without any definition of what is meant by the term. Nelson et al. [3] suggest that the concept of primary care is misused by chiropractors because they have not examined the concept in detail and thus do not understand it. Some of the confusion may lie in the varying definitions of primary care and primary care providers [4-6] and some may be due to a lack in understanding regarding the difference between the terms 'primary care' and 'portal of entry' or 'primary contact' (the term 'portal of entry' is often used in the US to describe a 'primary contact' practitioner healthcare providers) [7].

In order to determine whether or not a chiropractor should be considered as a primary healthcare practitioner, one must first attempt to define primary healthcare and then compare chiropractic practice with this definition.

There have been many definitions offered for the term primary care. One of the earliest and most influential is that proposed by Starfield [8] against which the attainment of primary care has often been measured. This definition of primary care focuses on four elements of care which need to be satisfied for a practitioner to be classed as a primary care practitioner. These are (i) first-contact, (ii) coordination of care, (iii) comprehensiveness and (iv) longitundinality (see Table 1).

**Table 1:** Criteria for defining primary care, according to Starfield [8].

*Aspect of primary care*	*Main elements of each aspect of care*
1. First-contact care	▪ Easy access (geographically and opening hours) ▪ Accessibility and utilisation by defined patient population
2. Coordination of care	▪ Scheduling arranged to allow patients to see the same primary care provider each visit ▪ Continuity of medical records ▪ 'Problem recognition' - follow up on status of previously identified problems at subsequent visits
3. Comprehensiveness	▪ Range of care services provided ▪ Services provided made explicit to the patient population ▪ Practitioners recognise a broad spectrum of needs within their patients
4. Longitudinality	▪ Patients identify the practice facility as their regular/main source of care over a period of time

Applying these criteria to chiropractic practice it is apparent that chiropractors usually provide a service that fulfils the requirements for coordination of care, and can readily achieve the requirements for first-contact by extending opening hours or providing an out of hours service. However, chiropractors may struggle to fulfil the other requirements. For example, the description of comprehensiveness states that practitioners must be able to arrange referral for all types of healthcare services, including supporting services such as home care and other community services; chiropractors do not usually have direct access to such services. Furthermore, the fact that the therapeutic scope of chiropractic is limited and that chiropractors do not have prescribing rights is often cited as a reason why chiropractors cannot provide the comprehensive services of primary care [9]. However chiropractors in some countries, such as Switzerland, already have limited prescribing rights [10] and others, including the UK, are considering it [11]. When considering longitudinality, patients need to identify the practice as their regular main source of healthcare; research shows that in chiropractic practice this is not the case [12-14].

More recently The American Academy of Family Physicians (AAFP) defined a primary care practitioner as *"a generalist physician who provides definitive care to the undifferentiated patient at the point of first contact and takes continuing responsibility for providing the patient's care." *[15]. In addition, the AAFP states that a *"primary care physician serves as the entry point for substantially all of the patient's medical and health care needs - not limited by problem origin, organ system, or diagnosis. Primary care physicians are advocates for the patient in coordinating the use of the entire health care system to benefit the patient."*. The term 'physician' as used in this definition, refers only to doctors of medicine or doctors of osteopathy. When comparing chiropractic practice to this definition of primary care, the limited therapeutic scope, as well as the lack of direct access to other aspects of the health care system, still obviates the 'primary care' claim.

The definition of primary care used by the Department of Health (DOH) in the United Kingdom (UK) is far less prescriptive, referring to primary care as the term for *"the health services that play a central role in the local community: GPs, pharmacists, dentists and midwives" *with primary care providers usually being the first point of contact for a patient and also following the patient through their care pathway [16]. In 2006 the DOH referred to chiropractors in the UK as Allied Healthcare Professionals (AHPs). They proposed that AHPs working within Clinical Assessment and Treatment Services (CATS) could develop capacity in primary care by offering a wider range of non-surgical alternatives for musculoskeletal conditions at the interface between primary and secondary care [17]. The phrase "primary healthcare" is now being used in reference to teams as opposed to individual practitioners, with the concept being that the primary healthcare team is *"dynamic rather than static, professional input changing to meet the changing needs of patients and groups of patients in different circumstances" *[18]. Chiropractors could therefore contribute to these primary healthcare teams, and the NICE Guidelines for the management of persistent non-specific low back pain, which include manual therapy and manipulation in its care pathway, could help facilitate this process [19]. Furthermore the recent white paper, Equity and excellence: Liberating the NHS [20], which sets out a plan to restructure the NHS including devolving power and responsibility for commissioning services to GP consortia, could generate opportunities for chiropractic services to be provided within the NHS.

When the principles of primary healthcare are examined in depth it can be seen that chiropractors could be considered to be primary healthcare professionals in some but not in all respects. This study aimed to determine the opinions of chiropractic educators and chiropractors in practice in the UK on the meaning of the use of the term primary healthcare when applied to chiropractic practice in the UK.

## Methods

This study formed part of a Doctoral thesis investigating the healthcare role of chiropractors in the UK from the viewpoint of chiropractic educators and chiropractors in practice. Prior to data collection full ethics approval was obtained from the Anglo-European College of Chiropractic (AECC) Ethics Sub-Committee. As the work was outside of the National Health Service (NHS), neither Local Research Ethics Committee (LREC) or Multi-centre Research Ethics Committee (MREC) approval were required.

**Figure 1:**
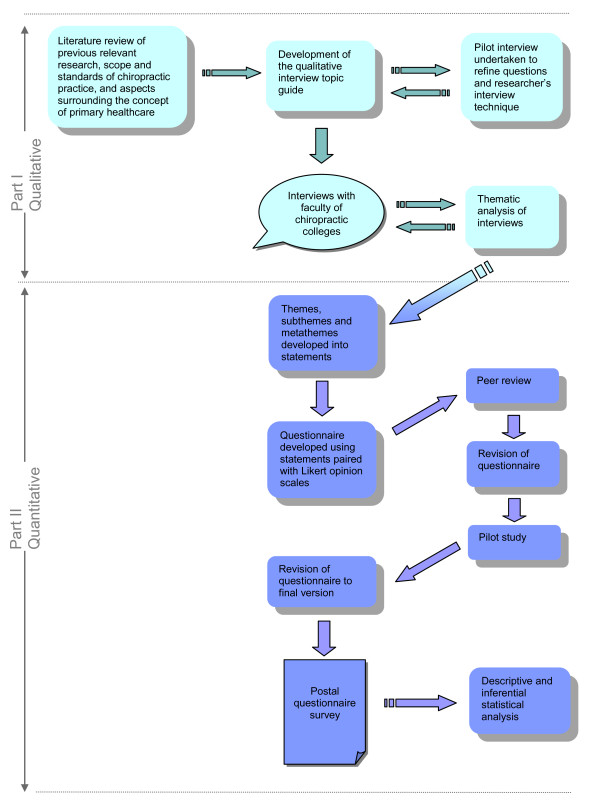
**Overview of the research methodology**.

The research implemented a sequential study of exploratory design. This model is characterised by an initial phase of qualitative data collection and analysis that precedes and informs the quantitative phase of data collection and analysis. The findings of the two phases of the study are then integrated during the interpretation phase at the end of the study [21]. The flow of this research process is outlined in Figure 1. Such use of the results from a qualitative study to inform a survey is said to enhance the sensitivity and accuracy of the survey questions [22]. The design can also be used to generalise qualitative findings to different samples [23], as well as to determine the distribution of a phenomenon within a chosen population [24].

The first part of the study consisted of one to one, semi-structured, interviews with chiropractors who teach on courses in the UK that lead to a European Council on Chiropractic Education (ECCE) accredited qualification (the ECCE is the accrediting body for chiropractic education in Europe and is recognised by the CCEI). This is the level of qualification to practise chiropractic that is recognised worldwide. At the time of this study there were only two institutions in the UK that were ECCE accredited; AECC and Welsh Institute of Chiropractic (WIOC). Chiropractors teaching at the McTimoney College of Chiropractic were therefore excluded from the current study.

One to one interviews were chosen over focus groups as their advantages were considered to outweigh their disadvantages for this study. Although focus groups can generate additional data through the interaction between group participants, familiarity can hinder disclosure and participants may have been less forthcoming with opinions that could be considered outside of the professional norm when they know or work with each other. Additionally, professional hierarchy can influence the degree of participation and inhibit those participants in sub-ordinate roles, with less authority or with less experience. Whilst focus group discussions can provide a breadth of topics they can also yield less in-depth exploration of topics by discussions moving on before everyone has had the opportunity to express their thoughts on an issue. In contrast, the one-to-one interview process can allow participants to express themselves honestly, without fear of professional recrimination or peer pressure to conform, and also gives every participant an equal chance to express their own personal accounts sufficient depth [25]. For these reasons, and because of the logistical issues involved in arranging a focus group that all participants could attend, interviews were chosen over focus groups as the method for data collection.

Members of chiropractic faculty were chosen for the interviews because of their breadth and depth of knowledge and experience in the topic under study, as well their privileged position of being able to influence the opinions of new graduates and hence shape the views and opinions of the future profession. Individual participants were chosen from the parent population to represent a level of diversity according to clinical experience, place of work and number of years in chiropractic education. All participants gave informed consent to participate in the study.

Participants were asked the open-ended question: "What does the term "primary healthcare" mean to you when applied to chiropractic in the UK?" and later prompted to explain why/how they had come to their opinion. Through verbatim transcription of the interviews, coding of the transcripts and thematic analysis of the results, this qualitative approach enabled the construct of a number of conceptual themes to represent the current opinions of the members of faculty involved. Interviews were conducted until data saturation, which occurred at the 7^th ^participant.

The themes, subthemes and metathemes constructed from the interviews were used to derive statements for the questionnaires used in the second part of the study. Four of these statements related to opinions regarding the use of the term "primary healthcare". Each statement was paired with a 5 point Likert response scale asking respondents to rank their opinion from strongly agree through to strongly disagree. Prior to using the questionnaire an assessment of its face validity was undertaken through peer review and a pilot study. The six chiropractors used for the pilot study were a convenience sample of local chiropractors in education that would have been excluded from the main study, but who were not interviewed for the first part of the study. Each of the pilot study participants had also been chiropractors in practice prior to becoming lecturers, or were still in practice part-time, and so were similar to the target population for the main enquiry. Each completed the questionnaire alone and then gave feedback in a one to one interview concerning face validity and general feedback on the questionnaire including their reasons for completing it, or not, had they received it in practice.

The questionnaire was amended further to the pilot study and was then used in a postal survey of chiropractors who had graduated from an ECCE recognised institution and who were registered with the General Chiropractic Council (GCC) as practicing full time in the UK. These inclusion criteria meant that McTimoney and McTimoney-Corley chiropractors were excluded from the study. There were 1690 chiropractors eligible to take part in the study. They were ordered using a random number generator and the first 600 were selected to participate in the study and sent the questionnaire together with a covering letter and stamped-addressed return envelope. The law of diminishing returns was used to time a repeat mailing to non-responders.

Data from the returned questionnaires were entered into a Microsoft Office Excel 2003 spreadsheet. The data were analysed both descriptively and to explore the relationships between the grouping and response variables. The descriptive statistics were calculated using Microsoft Excel and the inferential statistics using SPSS (Statistical Program for the Social Sciences, Version 15). Ranked responses, as obtained from the Likert scale, are ordinal data. Since this is not a true numerical scale, the use of mean averages is not considered appropriate [26] and the median value was therefore used as a measure of the central tendency. Since this study used a single score to measure an opinion, care must be taken in interpreting the results as the median value gives no indication of the distribution of the data, thus the pattern of the response (i.e. the frequency distribution) was also analysed [27].

To enable inferential statistical analysis to investigate any differences in opinion between chiropractors who graduated from different colleges and between chiropractors who had been in practice for varying amounts of time, the data for the grouping were recoded. The data regarding country of chiropractic college of graduation were recoded into two groups: 'Europe' and 'Other' and the data for the number of years in practice were recoded into: 5 or less years, 6-10 years, 11-20 years and greater than 20 years. To increase the robustness of the data when looking for differences in response to the opinion statements between these groups, the data from the Likert scales were collapsed and recoded to reflect whether respondents agreed with the statement (i.e. ticked "strongly agree" or "agree") or disagreed with the statement (i.e. ticked "disagree" or "strongly disagree"). For this part of the analysis, the responses from those respondents who ticked "Neutral/don't know" were excluded. Relationships between grouping variables and response variables were explored using Pearson's Chi-Squared tests for nominal data. This reflected the categorical nature of the grouping and response variables. Fisher's Exact test was used in instances where the numbers entered into the cells of the contingency tables were less than 5. Statistical significance was set at *p *< 0.05.

## Results

### Results of the qualitative study

The sample consisted of 7 participants. The clinical experience of the participants (time since graduation) ranged from 2 to 27 years and their experience teaching from 1 to 15 years. Between them they had a combined experience of teaching chiropractic of 46 years and a combined experience of clinical practice of 112 years. The length of each interview was dependent upon each participant's responses and lasted between 25 and 45 minutes.

All participants were in agreement that the term "primary healthcare" when applied to chiropractic in the UK means "primary contact" whereby chiropractors have the ability to see patients directly and without referral from a medical practitioner. The term "gatekeeper" and the phrase "portal of entry" were also used to describe this primary contact role.

Interview 2: *"we are portal of entry healthcare providers, meaning that a patient can come to us directly without referral from GP… beside portal of entry, I could also use the term primary contact… provider"*

Some participants went on to define what they felt were the requirements of a chiropractor as a primary contact practitioner. These requirements centred about the premise that chiropractors are able to diagnose patients and either suitably treat or refer, and therefore, that in order to fulfil this primary contact role, chiropractors need to be trained in diagnosis.

Interview 1: *"That requires chiropractors to have a modicum of training in diagnosis and be able to um understand the differing presentations of underlying disease um so that you can assess the likelihood of underlying disease or assess whether a condition or a presentation falls within your scope of practice, so therefore you can refer the patient to the appropriate healthcare professional whether it's their GP or specialist or someone else."*

Interview 3: *"I don't think we, we should hold ourselves up to be primary healthcare practitioners, I think we should be primary contact practitioners as in we know when to refer on and don't deal with everything."*

Some participants went further still to describe what they thought a primary healthcare practitioner was, and therefore why chiropractors do not fall under this category. The suggestion was that primary healthcare practitioners can diagnose and treat the most common medical conditions, and whilst chiropractors are able to diagnose many conditions they cannot *treat *all of the most common medical conditions. Hence, they should not be considered as primary healthcare practitioners.

Interview 2: *"Primary healthcare practitioner is a practitioner that can treat 60 of the most common medical conditions. So they can diagnose and treat 60 of the most common medical conditions. Which does not make chiropractors primary healthcare providers"*

One participant raised the question of whether primary healthcare is defined by role or by setting. This participant suggested that since the majority of chiropractors do not work in hospitals that they might be considered as primary healthcare professionals due to their location of practice outside of the hospital setting.

Interview 4: *"I talk about first contact but there's also this sense of being in, in a general practice environment for me rather than being in, in a hospital environment which I think of as being secondary."*

When participants were asked why they held these particular views on the healthcare role of chiropractors and what they understood by the term "primary healthcare", they described the development of opinions based upon undergraduate training, reinforced by experience, professional relationships and the GCC's code of practice and standards of proficiency.

Interview 5: *"my understanding of chiropractic when I came to the profession reinforced by my education, from being in private practice as well, GCC's… the role defined by the GCC, the role defined by most of the type of mission statements defining chiropractic as well"*

### Results of the quantitative study

**Figure 2:**
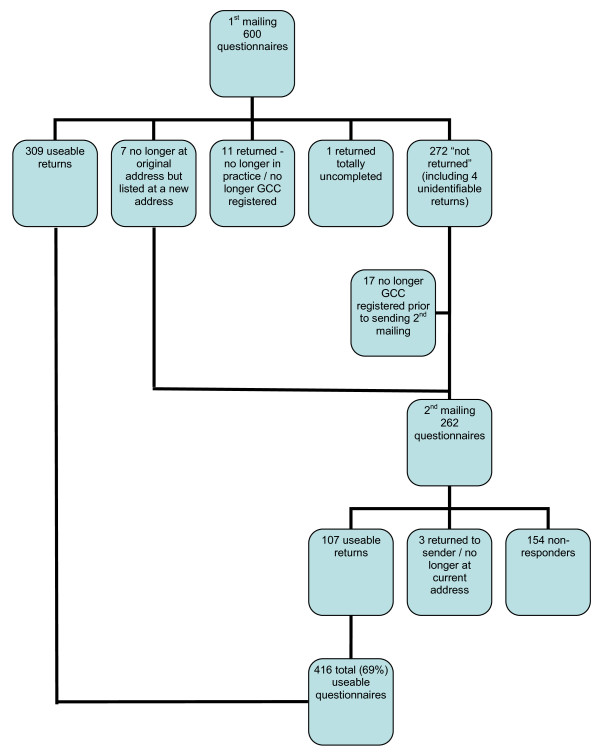
**Flow chart showing response to questionnaire mailings**.

A total of 416 useable questionnaires out of a possible 600 were returned, making an overall response rate of 69%. The response rate at each step of data collection is detailed in Figure 2. A total of 31 (5%) out of the 600 chiropractors sampled were known to be lost due to non-contact. Since the overall response rate was 69%, the total unit non-response was consequently 31% (5% due to non-contact and 26% due to refusal to participate).

**Figure 3:**
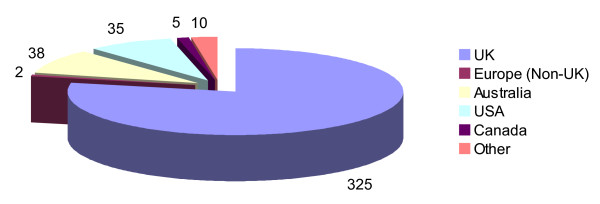
**Location of chiropractic college from which respondents graduated**. UK = United Kingdom; USA = United States of America.

Respondents graduated as chiropractors between the years of 1962 and 2006 with a mean average number of years in practice of 9.7 (SD 8.14) years. The majority of respondents were new or recent graduates with 41% (n = 165) having graduated within the previous 5 years and an additional 20% (n = 80) between 6 and 10 years ago. A majority of the respondents (79%, n = 325) had graduated from a chiropractic college in the UK, followed by Australia (10%, n = 38) and the USA (8%, n = 35) (see Figure 3). The distribution of the college of graduation in the respondents was very similar to that within the sample overall suggesting that there was no bias due to non-response in this respect.

Due to the random nature of the selection of the 600 participants it can be said that the sample is likely to be representative of chiropractors in clinical practice who graduated from an ECCE accredited college. However, unless non-response was also random, the respondents will not necessarily be representative of the population. Through comparison of respondents to the original sample an indirect evaluation of non-responder bias can be made, however, this can only be done for demographic data that are publically available, in this case only the location of chiropractic college of graduation from which the sample graduated. However, the high response rate obtained in this study makes this less of an issue.

**Figure 4:**
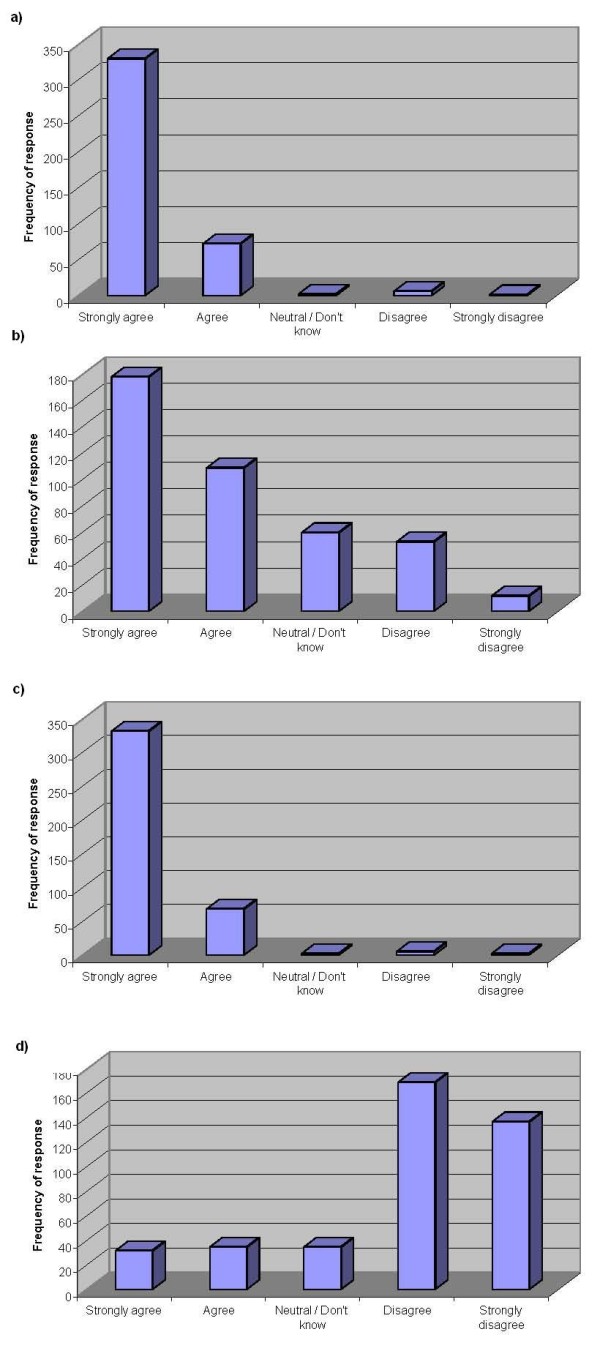
**Opinions of respondents regarding the chiropractic profession as one of "primary healthcare"**. (a) Responses to the statement: 'The term "primary healthcare", when applied to chiropractic in the UK, means primary contact' (n = 415) Median value = 'strongly agree' (b) Responses to the statement: 'The term "primary healthcare", when applied to chiropractic in the UK, means that chiropractors work in a primary healthcare setting' (n = 412) Median value = 'agree' (c) Responses to the statement: 'To be primary contact practitioners, chiropractors must be able to diagnose patients and refer when required' (n = 413) Median value = 'strongly agree' (d) Responses to the statement: 'The term "primary healthcare", when applied to chiropractic in the UK, means that chiropractors are able to treat all of the most common medical conditions' (n = 408) Median value = 'disagree'.

Four statements were provided to determine opinions of the respondents regarding the role of chiropractors as "primary healthcare" practitioners, each pertaining to a different aspect of "primary healthcare". The respondents were in agreement that when the term "primary healthcare" is applied to the chiropractic profession in the UK it means that chiropractors are primary contact practitioners (Figure 4a) who work in a primary healthcare setting (Figure 4b), and that in order to fulfil this role, chiropractors must be able to diagnose patients and make appropriate referrals to other healthcare professionals when necessary (Figure 4c). Respondents did not, however, consider that this entailed being able to treat all of the most common medical conditions that may present to them (Figure 4d).

The inferential data analysis was used to determine differences in responses to the statements between chiropractors that graduated from different educational institutions and between chiropractors that had been qualified for different periods of time.

This analysis revealed no statistically significant differences in opinion between chiropractors who graduated from European countries and those from other countries, nor between chiropractors who had graduated 5 or less years ago, 6-10 years ago, 11-20 years ago and greater than 20 years ago.

## Discussion

The results from both parts of this study are considered and interpreted collectively as is usual for a sequential study of exploratory design.

The results of this study show that there was consensus of opinion that chiropractors are primary contact practitioners (meaning having the ability to see patients directly and without the need for referral from a medical practitioner), who work in a primary healthcare setting and that central to this primary contact role is the ability to arrive at a diagnosis and refer patients when appropriate. Furthermore, the majority disagreed with the statement that chiropractors can treat all of the most common medical conditions.

The consensus of opinion amongst the participants, which suggests that chiropractors are primary contact and not primary healthcare professionals, is contrary to the evidence in the US research literature that there is division of opinion within the profession on this topic [6,28,29]. It does however, concur with another UK based study, which found 98% of respondents considered a chiropractor to be a primary contact practitioner [30].

The criteria used to define primary healthcare in the USA are more strict and explicit in the USA compared to the UK suggesting that it could be easier for a chiropractor to claim a primary healthcare role in the UK than in the USA. However due to the differences in the healthcare systems there are other benefits, such as remunerations, to being classed as primary healthcare professionals in the USA and this may encourage chiropractors there to pursue a primary healthcare title. This might explain the apparent difference in opinion seen in the research literature between different countries. However, there was no significant difference in opinion found in this study between chiropractors that qualified in Europe compared with those that qualified elsewhere. It therefore seems that where a chiropractor practices is more influential on opinion regarding their healthcare role than is the country in which they were educated.

There was general agreement within this study that in order to fulfil a primary contact role a chiropractor must be able to diagnose patients and make referrals when a condition is not within their therapeutic scope of practice. This is in accordance with educational standards [31] and in line with legislation [1], but is in contrast to the findings of a survey of US chiropractic students by McCoy et al. in 2007, where only 58% felt that chiropractors have a duty to diagnose their patients even though 86.6% of them know that their state laws contained a "duty to diagnose" statement [7]. However, the study by McCoy et al. was of only one chiropractic college in the US and therefore is unlikely to be representative of the views of all students, let alone all chiropractors, in the USA.

### Limitations to the study

As with any research study, this study had a number of limitations. Some limitations are inherent to the research design itself; this is particularly the case with regard to the issue of reflexivity in the qualitative study. Due to the reflexive relationship between the researcher and the decontextualised data it is imperative that the researcher makes every effort to suspend any of their own pre-conceived ideas and to be open to the data as it is collected and analysed [32]. The researcher is inextricably woven into the process of data collection and analysis, and despite all best efforts to limit the impact of their own views, their own positionality inevitably influences the process to some degree. All interviews can also be influenced by the relationship and rapport between researcher and participant. An additional consideration in this study was that all but one of the interviewees knew the researcher. It has been suggested in the literature that as an insider the researcher is more likely than an outsider to obtain consent and willingness to participate [33]. Conversely, participants could have been inhibited in response if they did not want to reveal their true feelings to the researcher, or if they thought that this might affect the researcher's opinion of them. The author's impression was that the interviews with those participants known to her were open and honest, with the discourse flowing easily between researcher and participants.

A limitation of the qualitative data analysis was that it was completed by only one person (the researcher). Uncoded transcripts would ideally be given to a peer to code and analyse, and then both sets of analysed data would be re-examined for agreements and disagreements with subsequent review of categorise if needed. This can help verify the thematic analysis. However, due to the time-consuming nature of the coding and analysis of qualitative data it is often difficult to involve another researcher, as was the case in this study.

An inherent disadvantage of a questionnaire as a data collection tool is its unidirectional nature [34]. Postal questionnaires also have no ability to assess the honesty of responses [35]. Even though the covering letter and the questionnaire used in this study implored participants to give their personal and honest opinion, as well as emphasising anonymity and confidentiality, there was still the risk that participants could give responses they thought were the most professionally appropriate.

Not all aspects of primary care were considered in this study and this could also be considered a weakness. Co-ordination and longitudinality of care were not mentioned during the qualitative interviews and therefore, due to the study design, were not included in the questionnaire used for the survey.

### Generalisability of results

External validity, or generalisability of the findings of a study, is the concept that the findings are applicable to a wider population than the study sample. The main limitation of the current study with respect to this was the decision to exclude McTimoney and McTimoney-Corley chiropractors. These chiropractors are unique to the UK so including them in the study would have made comparisons with the relevant existing chiropractic research difficult, since much of it has been undertaken in the US, Canada and Australia. However, as a consequence of this decision, the results of the current study have limited generalisability within the UK itself as at the time of the survey McTimoney and McTimoney-Corley chiropractors together represented 22% of GCC registered chiropractors.

## Conclusions

Whether or not chiropractors are considered primary healthcare professionals is considered by some as central to the identity of the profession, and entwined with scope of practice. Most of the research debating this however has come from the US, Canada and Australia; little research existed on the opinions of chiropractors in the UK towards the healthcare role they play. This study aimed to inform this subject through a sequential study of exploratory design.

The findings of this study suggest that chiropractors in the UK view their role as one of a primary contact healthcare practitioner. Chiropractors are also cognisant of the fact that their primary contact status means that they must be able to diagnose and refer conditions outside their therapeutic scope of practice. This is inline with the DOH definition of an AHP and this clarity should help define the role of chiropractors within the healthcare community in the UK.

Contrary to popular opinion, the results of this study suggest there is little disagreement between chiropractors in the UK regarding their primary contact healthcare role. There were also no major differences in opinion between chiropractors who qualified in Europe and those who qualified in non-European countries on any of the issues surveyed. This suggests that the differences in findings between the current study and those reported in the literature for chiropractors practising in the US may be more associated with the context of practice (i.e. country) rather than the country they graduated from. This implies that a unified view of professional identity and healthcare role is perhaps more likely within individual countries than can be achieved on an international basis. This may be due to the differences in the needs of the healthcare systems in different countries that will, to some degree, determine the healthcare role that chiropractors need to fulfil.

Further studies need to be undertaken including an evaluation of the current research within a wider healthcare context. In particular the opinions of other healthcare professionals, towards the role of chiropractors in healthcare needs to be examined in more detail. This would enable a comparison of how the chiropractic profession in the UK views itself and how it is viewed by orthodox medicine, which could help facilitate better interprofessional relationships. A similar study of those chiropractors that were excluded from the current study would also be of interest.

Chiropractors in the UK are currently in the fortunate position of being primary contact healthcare providers. As the profession becomes increasingly regulated as reflected by the formation of the GCC and more recently the Council for Healthcare Regulatory Excellence (CHRE), it is imperative that the profession maintains autonomy and precludes limitation of practice, whilst simultaneously developing closer links with other healthcare professionals and promoting interdisciplinary care including becoming more involved with primary healthcare teams within the NHS.

## List of abbreviations

AAFP: The American Academy of Family Physicians; AECC: Anglo-European College of Chiropractic; AHP: Allied Healthcare Professional; CATS: Clinical Assessment and Treatment Services; CCEI: Council for Chiropractic Education International; CHRE: Council for Healthcare Regulatory Excellence; CHRP: Council for the Regulation of Healthcare Professionals; DOH: Department of Health; ECCE: The European Council on Chiropractic Education; GCC: General Chiropractic Council; GP: General Practitioner; LREC: Local Research Ethics Committee; MREC: Multi-centre Research Ethics Committee; NHS: National Health Service; NICE: National Institute for Health & Clinical Excellence; SPSS: Statistical Program for the Social Sciences; UK: United Kingdom; US: United States; USA: United States of America; WIOC: Welsh Institute of Chiropractic.

## Competing interests

The author is a chiropractor and a full-time, senior lecturer at the Anglo-European College of Chiropractic.

## Authors' contributions

AJH designed the research, performed the literature search, carried out the data collection and analysis, and wrote the manuscript. The research findings reported in this manuscript form part of a larger research thesis undertaken by AJH in completion of a Professional Doctorate.
